# A sixteen decimal places' accurate Darcy friction factor database using non-linear Colebrook's equation with a million nodes: A way forward to the soft computing techniques

**DOI:** 10.1016/j.dib.2019.104733

**Published:** 2019-10-28

**Authors:** Muhammad Mujtaba Shaikh, Shafiq-ur-Rehman Massan, Asim Imdad Wagan

**Affiliations:** a Department of Basic Sciences and Related Studies, Mehran University of Engineering and Technology, Jamshoro, Sindh, Pakistan; b Supply Chain and Operations Management Research Group, Mehran University of Engineering and Technology, Jamshoro, Pakistan; c Shaheed Zulfikar Ali Bhutto Institute of Science and Technology, Sindh, Pakistan; d Mohammad Ali Jinnah University, Karachi, Sindh, Pakistan

**Keywords:** Colebrook's equation, Darcy friction, Soft computing, Accurate database, Nonlinear equation

## Abstract

The Colebrook's equation is considered as an empirical model to accurately compute the Darcy friction factor in pipes under fully-developed turbulent flow. Due to non-linearity and implicitness of the Colebrook's equation, one needs to use numerical methods to acquire reasonably good approximation to the true friction factor values. However, such idea is not preferred by practitioners as it demands use of computers – also more computational time and effort. To overcome this, explicit equations that can describe Darcy friction factor directly in terms of the Reynolds number and relative roughness are essential. Using Fixed point iteration method in the MATLAB software, we have developed a 16 decimal places' accurate friction factor database for the Darcy friction factor for a 1000 by 1000 mesh of Reynolds number and relative roughness values. The accurate dataset described in this work will serve to be basis for the construction of new and more reliable explicit equations using regression modeling, artificial intelligence techniques and other soft computing methods.

**Table:** Specifications Table

Subject area	*Applied Numerical Mathematics; Mechanical, Civil, Hydraulic, Chemical, Industrial Engineering*
More specific subject area	*Single and Multi-phase pipe flow*
Type of data	*Tables and excel file*
How data was acquired	*Numerical method on non-linear Colebrook's equation*
Data format	*Raw and analyzed*
Experimental factors	*A numerical method – Fixed point iteration method – has been used to compute the friction factor data corresponding to million nodes for Reynolds number and pipe relative roughness.*
Experimental features	*Friction factor varies with respect to the Reynolds number and relative roughness of pipes, and it has been computed and analyzed using 1000 by 1000 square mesh for Reynolds number and relative roughness values. The ready data available with this article helps practitioners quickly know the friction factor for any particular value of Reynolds number and pipe's relative roughness.*
Data source location	*The accurate data shared in this article was computed numerically using Fixed point iteration method with experimentally used 1000 by 1000 values of relative roughness and Reynolds numbers from literature at SMMK office, Department of Basic Sciences and Related Studies, Mehran University of Engineering and Technology, Jamshoro, Sindh, Pakistan [25.4084° N, 68.2605° E]*
Data accessibility	*Data is available as supplementary material to this article.*

**Table:** 

**Value of the Data** • The Data presented in this article provides sixteen decimal places' accurate Darcy friction factor profile versus a combination of million relative roughness and Reynolds number values. • This data is primarily beneficial for practitioners in the field working with pipe flow dynamics to directly get the Darcy friction factor values from the supplementary material instead of using any nonlinear solver or explicit equation to approximate the friction value. • The data shared in this article will benefit forthcoming studies in this field as the ready database in this article, with double precision accuracy, can directly be used as basis for soft computing techniques to propose new explicit equations to approximate friction factor. • The present database can be used to verify any other method – be it analytical, numerical or empirical – to check the accuracy of computed values. • For more comprehensive studies related to single and multi-phase pipe flow systems, pipe friction factor is a basic requirement. • Using the data given in this paper highly accurate estimate to energy and pressure losses in rough pipe networks can be obtained under fully developed turbulent flow. • Present Data can be used in future for the proposal of more accurate explicit equations for the Darcy friction factor modeling using soft computing techniques, like *regression modeling* [1,2]*, gene programming* [3,4], *artificial neural networks* [[5], [6], [7]] *and adaptive neuro-fuzzy inference system* [[8], [9], [10]].

## 1 Data

The Colebrook's equation nonlinearly relates the friction factor in a pipe fluid flows. For particular cases, and with some specified values of relative roughness and the Reynolds number, the nonlinear equation can be solved numerically for the friction factor.

To overcome the complexities due to numerical methods, explicit approximations are usually preferred. The explicit approximations can be developed using accurate database of friction factor values from the Colebrook's equation.

The data in this work comprises of the sixteen decimal places' accurate friction factor solution of the nonlinear Colebrook's equation for a range wider range of values of relative roughness and Reynolds number, thousand both; thus in total a million nodes. A part of this data (see supplementary material) has been used in Ref. [11] to propose a new and efficient explicit approximation to find the friction factor approximation using a pair of known values of relative roughness and Reynolds number.

## 2 Experimental design, materials and methods

The Colebrook's equation [[1], [2], [3], [4], [5], [6], [7], [8], [9], [10], [11]] is given as:

(1) $$\frac{1}{\sqrt{z}}=-2\;\log\left(\frac{2.51}{x\sqrt{z}}+\frac{y}{3.7}\right)$$

where *z* is the Darcy friction factor, *x* is the Reynolds number and *y* is the relative roughness.

Using numerical methods, to any desired accuracy, one can solve equation (1) for *z* for a given pair (*x*, *y*).

Using a 1000 by 1000 mesh of values of *x* and *y*, defined as:

(2) $$\text{x: }4000\text{: }99996\text{: }10^{8}$$

(3) y: 0: 0.00005: 0.05

An accurate database can be obtained, particularly to an accuracy of 16 decimal places, using Fixed-Point iteration method [11] starting with an initial assumption $z^{\left\{0\right\}}=0.1$ as given in equation (3).

(4) $$z^{\left\{i+1\right\}}=0.25{\left[\log\left(\frac{2.51}{x\sqrt{z^{\left\{i\right\}}}}+\frac{y}{3.7}\right)\right]}^{-2}\;\text{for , i }=\text{ 0,}1\text{,}2..$$

Usually after 30 repeated evaluations of (4) i.e. 30 iterations as used in Ref. [11], we can get 16 decimal places accurate numerical solution to (1), however the present data has been obtained upto 50 iterations for more careful examination.

After getting the dataset i.e. values of *z* (a matrix of 1000 by 1000 nodes based on distribution of *x* and *y*), soft computing techniques like: regression modeling, artificial neural networks and adaptive neuro-fuzzy inference system can be employed to get best explicit relationships of *z* in terms of *x* and *y*, like:

(5) z=f(x,y)

The obtained dataset has been generated in MATLAB software with double precision arithmetic using the Fixed point iteration method (4). With an initial guess of 0.1, and continuing for more than fifty iterations the sixteen decimal places' accurate solutions to the Colebrook's equation have been obtained for a million nodes.

The dataset is attached as supplementary material to this article in form of a Microsoft Excel file. The first three sheets, respectively, contain million values of relative roughness (2), Reynolds number (3) and the consequent Darcy friction factor values (4). The breakup is best described in Table 1.

**Table 1:** Layout and breakup of the dataset included as supplementary material.

Sheet 1 (Reynols numbers) INPUT *x*	Sheet 2 (Relative Roughness) INPUT *y*	Sheet 3 (accurate friction factors) OUTPUT *z*	Sheet 4 (validity of the data) *F* (*x*, *y*; z*)
1000 by 1000 values [*x*^*T*^*x*^*T*^*… x*^*T*^] using (2)	1000 by 1000 values [*y*^*T*^*y*^*T*^*… y*^*T*^] using (3)	1000 by 1000 values [*z*^*T*^*z*^*T*^*… z*^*T*^] using (4) 50 times	1000 by 1000 values [*F*^*T*^*F*^*T*^*… F*^*T*^] using (7) 50 times

## 3 Verification of accuracy of the proposed database

It is important to verify how the obtained database is accurate, and there can be many ways of checking this. The most obvious, straightforward and the primary way to investigate the 16 decimal places’ accuracy of the computes friction factor values if the database is to check these through the actual equation – the Colebrook equation (1). For example [12], if one claims that

x∗= -1.207647827130918927009

is a solution of the nonlinear equation (6),

(6) $$G\left(x\right)=xe^{x^{2}}-\sin^{2}\;x+3\;\cos\;x+5=0$$

then the validity of the claim for x* can be checked by substituting claimed x* in (6), i.e. by checking if *G* (x*) = 0.

Similarly, accuracy check has been performed to investigate the claimed double precision accuracy of the computed database of solutions *z*'s (see supplementary material) of the nonlinear Colebrook's equation (1) for 1000 by 1000 values of Reynolds number (*x*'s) and relative roughness (*y*'s). Re-writing (1) in the form:

(7) $$F\left(x,y;z\right)=\frac{1}{\sqrt{z}}+2\;\log\left(\frac{2.51}{x\sqrt{z}}+\frac{y}{3.7}\right)=0$$

the used values of inputs *x* and *y* (from sheets 1 and 2 of the supplementary file, respectively) and the computed accurate values of output *z* (from sheet 3 of the same file) have been substituted in (7) and for the million nodes *F* (*x*, *y*; z*) have been analyzed, which are available on sheet 4 of the supplementary file. For all million nodes of Reynolds number and pipe relative roughness, the values of *F* (*x*, *y*; z*) have been found to be exactly equal to 0 or 1E-16 (to double precision default in Ms Excel). The values on sheet 4 of the supplementary file confirm the claimed accuracy of the presented database of the Darcy friction factor values (*z*'s) corresponding to *x*'s and *y*'s. It should be noted that any other way of investigating the accuracy of database would be a secondary analysis, and mostly in the context of such numerical calculations the examination of the equality in the nonlinear equation which has been solved at the claimed solution is given top priority, see Refs. [11,12].

The presented database is a standalone accuracy checker of any explicit equation in literature, for example 16 decimal places’ accurate friction factor values of this database (see Sheet 3 of the supplementary material) has been used successfully in Ref. [11] to verify the accuracy of a new explicit equation proposed therein by us and a few others by Manadilli [13], Fang et al. [14] and Brkić [15].

The dataset can be used in future for proposition of robust and highly accurate explicit approximations for the Darcy friction factor by means of many soft computing techniques, and thus, will provide a way forward for the mathematicians, engineers and data scientists in analysis of pipe flow systems. On the other hand, the ready dataset can be readily used by practitioners to know the friction factor exactly for different values of relative roughness and Reynolds numbers.
